# Association Between Birth Weight and Risk of Pregnancy-Induced Hypertension and Gestational Diabetes in Japanese Women: JPHC-NEXT Study

**DOI:** 10.2188/jea.JE20200302

**Published:** 2022-04-05

**Authors:** Kohei Ogawa, Naho Morisaki, Aurelie Piedvache, Chie Nagata, Haruhiko Sago, Kevin Y. Urayama, Kazuhiko Arima, Takayuki Nishimura, Kiyomi Sakata, Kozo Tanno, Kazumasa Yamagishi, Hiroyasu Iso, Nobufumi Yasuda, Tadahiro Kato, Isao Saito, Atsushi Goto, Taichi Shimazu, Taiki Yamaji, Motoki Iwasaki, Manami Inoue, Norie Sawada, Shoichiro Tsugane

**Affiliations:** 1 Center for Maternal-Fetal, Neonatal and Reproductive Medicine, National Center for Child Health and Development, Tokyo, Japan; 2 Department of Social Medicine, National Research Institute for Child Health and Development, Tokyo, Japan; 3 Department of Education for Clinical Research, National Center for Child Health and Development, Tokyo, Japan; 4 Graduate School of Public Health, St. Luke’s International University, Tokyo, Japan; 5 Department of Public Health, Nagasaki University Graduate School of Biomedical Sciences, Nagasaki, Japan; 6 Department of Hygiene and Preventive Medicine, School of Medicine, Iwate Medical University, Iwate, Japan; 7 Department of Public Health Medicine, Faculty of Medicine, and Health Services Research and Development Center, University of Tsukuba, Ibaraki, Japan; 8 Ibaraki Western Medical Center, Ibaraki, Japan; 9 Public Health, Department of Social Medicine, Osaka University Graduate School of Medicine, Osaka, Japan; 10 Department of Public Health, Kochi University Medical School, Kochi, Japan; 11 Center for Education and Educational Research, Faculty of Education, Ehime University, Ehime, Japan; 12 Department of Public Health and Epidemiology, Oita University, Oita, Japan; 13 Epidemiology and Prevention Group, Center for Public Health and Sciences, National Cancer Center, Tokyo, Japan

**Keywords:** gestational diabetes mellitus, lower birth weight, pregnancy induce hypertension, transgenerational effect

## Abstract

**Background:**

Although prevalence of low birth weight has increased in the last 3 decades in Japan, no studies in Japanese women have investigated whether birth weight is associated with the risk of pregnancy complications, such as pregnancy-induced hypertension (PIH) and gestational diabetes mellitus (GDM).

**Methods:**

We used data from the Japan Public Health Center-based Prospective Study for the Next Generation (JPHC-NEXT), a population-based cohort study in Japan that launched in 2011. In the main analysis, we included 46,365 women who had been pregnant at least once, for whom information on birth weight and events during their pregnancy was obtained using a self-administered questionnaire. Women were divided into five categories according to their birth weight, and the relationship between birth weight and risk of PIH and GDM was examined using multilevel logistic regression analyses with place of residence as a random effect.

**Results:**

Compared to women born with birth weight of 3,000–3,999 grams, the risk of PIH was significantly higher among women born <1,500 grams (adjusted odd ratio [aOR] 1.60; 95% confidence interval [CI], 1.17–2.21), 1,500–2,499 grams (aOR 1.16; 95% CI, 1.03–1.30), and 2,500–2,999 grams (aOR 1.13; 95% CI, 1.04–1.22). The risk of GDM was significantly higher among women born 1,500–2,499 grams (aOR 1.20; 95% CI, 1.02–1.42), albeit non-significant association among women in other birthweight categories.

**Conclusions:**

We observed an increased risk of PIH among women born with lower birth weight albeit non-significant increased risk of GDM among Japanese women.

## INTRODUCTION

Studies show that the influence of being born with low birth weight has non-negligible influence on the individual. It increases the risk of infant and child mortality and morbidity including allergies and mental disorders, and recent studies suggest long-term influences that persist into adolescence and adulthood. Large epidemiological studies have shown robust evidence that being born with low birth weight is associated with risk of chronic disease, such as hypertension, diabetes mellitus, and coronary artery disease in later life.^1^^–^^4^ Additionally, several studies in Europe and the United states have reported associations between being born with lower birth weight and subsequent increased risk of preeclampsia^5^^–^^8^ and gestational diabetes^9^^–^^12^ during pregnancy. Thus, being born small may have a negative impact on not only their own health but the next generation’s health, as it is well known that fetuses that are exposed to maternal pregnancy complications in utero have higher risk of metabolic disease in later life.^13^^–^^16^

However, all studies to date have primarily been in populations of European ancestry, even though there may be differences in effect size by race.^8^^,^^17^^–^^19^ Japan has seen a unique trend compared to other countries, as prevalence of low birth weight has increased in the last three decades.^20^ Investigating the extent to which being born with lower birth weight influences the risk of pregnancy complications would be of great value to understand the population burden of the observed increases in low birth weight. Thus, we targeted a population of Japanese women within the context of a large Japanese cohort study and examined the association between birth weight and risk of pregnancy complications, namely pregnancy-induced hypertension (PIH) and gestational diabetes (GDM).

## METHODS

### Study population

This analysis utilized data from the Japan Public Health Center-based Prospective Study for the Next Generation (JPHC-NEXT), a population-based cohort study launched in 2011 comprising seven prefectures of Japan with the main aim to elucidate the risk factors for lifestyle-related disease and contribute to the development of personalized healthcare.^21^ Briefly, the JPHC-NEXT study considered 261,939 men and women aged 40–74 years registered into the resident registration system of the seven target prefectures as its target population. Self-administered questionnaires were distributed to this population; participants were also asked to participate in bio-specimen collection and longitudinal follow-up. The questionnaire included items asking about their own birth weight, pregnancy history, medical history as well as lifestyle. Among 61,539 women who completed the questionnaire, 55,303 women had given birth at least once (after excluding 18 women who responded having their first child under the age of 10 years). After exclusion of participants who did not provide their birth weight, 46,365 women remained for the main analysis.

All participants gave their written informed consent at the time of recruitment. The study protocol was approved by the Institutional Review Board at the National Cancer Center on April 11, 2018 (No. 2017-250) as well as at each collaborating institution of the regional areas. The analysis protocol was approved by the Institutional Review board at the National Center for Child Health and Development on June 4, 2018 (No. 1847).

### Measures

Two major complications related to pregnancy, PIH and GDM, were considered as the primary outcomes for this study. Data on self-reported medical history of PIH and GDM (Have you experienced hypertensive disorders in pregnancy (pregnancy-induced hypertension, preeclampsia)? yes/no; Have you experienced eclampsia? yes/no; Have you ever been told that your blood sugar is high while you were pregnant? yes/no) were collected from the questionnaires.

The primary exposure of interest was self-reported birth weight with possible response categories including: <1,500 grams, 1,500–2,499 grams, 2,500–2,999 grams, 3,000–3,999 grams, and >4,000 grams. Possible confounding variables considered when examining the effect of birth weight on risk of pregnancy complications were age at first pregnancy, height (quartile cut-points), body mass index (BMI) at 20 years (<18.5, 18.5–24.9, or ≥25.0 kg/m^2^), family history of diabetes and hypertension (father or mother), having an older brother or sister (none, at least one, or more than one), and educational attainment (junior high school; high school; vocational school, college; or 4-year university or higher), according to previous studies.^8^^,^^10^ Additionally, we considered smoking status at two timepoints as potential confounding factors; maternal smoking at first pregnancy and passive smoking around 10 years old (almost none; 1–3 times a month; 1–4 times a week; almost every day). As the participants had a wide range of birth years and place of residences for which prevalence of our outcomes could have varied, we considered those as confounders as well.

### Statistical analysis

Multilevel logistic regression analyses was performed with place of residence (seven prefectures) as the random effect to estimate the association between birth weight and odds of pregnancy complications. To examine the influence of confounding by various co-variables, four nested models were considered. We tested for independence between confounders upon including all variables and included an interaction term when significant interaction was present (interaction term had *P* value less than 0.25).^22^ The base model (Model 1) included birth year considering potential influence on both birth weight and outcome. In model 2, we included demographics of the mother as potential confounders. Since we did not have direct information regarding the mothers of the pregnant women except for family history of hypertension, we used the following variables as proxies: number of siblings as proxy for the mother’s parity, height of women as a proxy for height of the mothers, passive smoking status as a child as proxy of the mothers smoking status during pregnancy, and education as proxy of the mothers social economic status during pregnancy. The third model (Model 3a) additionally considered factors (and their proxies) of the pregnant women known to be strongly related to pregnancy outcomes: age at first pregnancy as proxy for age at pregnancy, and current smoking status as proxy for smoking status during pregnancy. For this model, interaction between age at first pregnancy and birth year were included as the interaction was statistically significant. Finally, the last model (Model 3b) included BMI at age 20 years to evaluate whether this may play a mediating role in the association between birth weight and pregnancy complications.

We conducted our main analysis on women who had self-reported birth weight and at least one pregnancy (excluding 8,938 women who had missing values on self-reported birth weight). There were moderate levels of missing data for other variables including passive smoking (8.6%), BMI at age 20 years (5.5%), education (1.9%), age at first delivery (0.9%), and height (0.7%); thus, we performed imputation on all variables except for birth weight and refer to this as the partially imputed dataset (*n* = 46,365). We performed multiple imputation by chained equation on 25 datasets for which the completed analysis results were consolidated into one inference using Rubin’s combination rules.^23^ Two sensitivity analyses were conducted. Because missing birth weight data was not negligible and differed by birth weight (imputed results suggested those born with more extremely high or low birth weights were more likely to remember their birth weight category), the first was conducted on a fully imputed dataset where all variables, including birth weight, were imputed among all women who had a pregnancy (*n* = 55,303). To confirm the robustness of the primary analysis, the second was conducted among all women who had a pregnancy with no missing data for all variables considered in the analysis (*n* = 41,285).

We performed all statistical analyses using the statistical software package, Stata SE 15 (STATA Corp, College Station, TX, USA), and considered a two-sided *P*-value of less than 0.05 to be statistically significant.

## RESULTS

We considered 46,365 women who had a pregnancy and provided data on their birth weight. Maternal demographics by each category of birth weight are shown in Table 1. For all variables, significant differences were observed by birth weight category. Distribution of demographic variables after multiple imputation for fully imputed (*n* = 55,303) datasets as well as those without any missing data (*n* = 41,285) are shown in eTable 1.

**Table 1.** Distribution of maternal demographics by categories of birth weight among women who had a pregnancy

	Total (*N* = 55,303)	Missing on birth weight data (*n* = 8,938)	Women with birth weight data (*n* = 46,365)	Birth weight					*P*-value^c^

				<1,500 g (*n* = 365)	1,500–2,499 g (*n* = 5,088)	2,500–2,999 g (*n* = 26,099)	3,000–3,999 g (*n* = 14,498)	>4,000 g (*n* = 315)	
	*N*	*n* (%)	*n*	*n* (%)	*n* (%)	*n* (%)	*n* (%)	*n* (%)	
**Birth year**									
1936–1945	11,149	3,741 (33.6)	7,408	95 (1.3)	1,087 (14.7)	4,846 (65.4)	1,362 (18.4)	18 (0.2)	<0.001
1946–1955	20,720	4,193 (20.2)	16,527	155 (0.9)	2,109 (12.8)	10,772 (65.2)	3,447 (20.9)	44 (0.3)	
1956–1965	14,484	850 (5.9)	13,634	80 (0.6)	1,195 (8.8)	6,964 (51.1)	5,298 (38.9)	97 (0.7)	
1966–1977	8,950	154 (1.7)	8,796	35 (0.4)	697 (7.9)	3,517 (40.0)	4,391 (49.9)	156 (1.8)	
**Age at first pregnancy, years**									
10–24	22,150	4,493 (20.3)	17,657	154 (0.9)	2,064 (11.7)	10,534 (59.7)	4,820 (27.3)	85 (0.5)	<0.001
25–29	25,120	3,627 (14.4)	21,493	167 (0.8)	2,307 (10.7)	12,071 (56.2)	6,806 (31.7)	142 (0.7)	
30–34	5,669	533 (9.4)	5,136	33 (0.6)	518 (10.1)	2,531 (49.3)	1,991 (38.8)	63 (1.2)	
≥35	1,829	165 (9.0)	1,664	10 (0.6)	154 (9.3)	741 (44.5)	736 (44.2)	23 (1.4)	
Missing	535	120 (22.4)	415	1 (0.2)	45 (10.8)	222 (53.5)	145 (34.9)	2 (0.5)	
**BMI at 20 years, kg/m^2^**									
<18.5	6,540	685 (10.5)	5,855	63 (1.1)	827 (14.1)	3,226 (55.1)	1,707 (29.2)	32 (0.5)	<0.001
18.5–24.9	42,595	5,916 (13.9)	36,679	262 (0.7)	3,809 (10.4)	20,843 (56.8)	11,521 (31.4)	244 (0.7)	
≥25.0	3,140	631 (20.1)	2,509	29 (1.2)	298 (11.9)	1,314 (52.4)	846 (33.7)	22 (0.9)	
Missing	3,028	1,706 (56.3)	1,322	11 (0.8)	154 (11.6)	716 (54.2)	424 (32.1)	17 (1.3)	
**Having an older brother or sister**									
No	19,791	2,468 (12.5)	17,323	129 (0.7)	1,989 (11.5)	9,507 (54.9)	5,585 (32.2)	113 (0.7)	<0.001
One	15,787	2,100 (13.3)	13,687	95 (0.7)	1,329 (9.7)	7,308 (53.4)	4,835 (35.3)	120 (0.9)	
More than one	19,725	4,370 (22.2)	15,355	141 (0.9)	1,770 (11.5)	9,284 (60.5)	4,078 (26.6)	82 (0.5)	
**Maternal height, cm**									
<151	14,034	3,600 (25.7)	10,434	158 (1.5)	1,885 (18.1)	6,769 (64.9)	1,605 (15.4)	17 (0.2)	<0.001
151–155	16,712	2,769 (16.6)	13,943	121 (0.9)	1,598 (11.5)	8,508 (61.0)	3,670 (26.3)	46 (0.3)	
156–159	12,947	1,508 (11.6)	11,439	53 (0.5)	961 (8.4)	6,209 (54.3)	4,125 (36.1)	91 (0.8)	
≥160	11,226	843 (7.5)	10,383	33 (0.3)	613 (5.9)	4,522 (43.6)	5,054 (48.7)	161 (1.6)	
Missing	384	218 (56.8)	166	0 (0.0)	31 (18.7)	91 (54.8)	44 (26.5)	0 (0.0)	
**Family history of DM**	8,347	954 (11.4)	7,393	57 (0.8)	785 (10.6)	3,912 (52.9)	2,560 (34.6)	79 (1.1)	<0.001
**Family history of HTN**	18,277	2,255 (12.3)	16,022	107 (0.7)	1,702 (10.6)	8,811 (55.0)	5,288 (33.0)	114 (0.7)	<0.001
**Passive smoking at 10 years**									
Almost none	25,862	4,105 (15.9)	21,757	156 (0.7)	2,364 (10.9)	12,548 (57.7)	6,554 (30.1)	135 (0.6)	<0.001
1–3 times a month	2,067	189 (9.1)	1,878	8 (0.4)	191 (10.2)	1,035 (55.1)	625 (33.3)	19 (1.0)	
1–4 times a week	4,139	394 (9.5)	3,745	27 (0.7)	336 (9.0)	2,094 (55.9)	1,263 (33.7)	25 (0.7)	
Almost every day	18,493	2,582 (14.0)	15,911	120 (0.8)	1,766 (11.1)	8,556 (53.8)	5,346 (33.6)	123 (0.8)	
Missing	4,742	1,668 (35.2)	3,074	54 (1.8)	431 (14.0)	1,866 (60.7)	710 (23.1)	13 (0.4)	
**Smoking status at first pregnancy** ^a^									
No	48,534	8,407 (17.3)	40,127	319 (0.8)	4,454 (11.1)	23,109 (57.6)	11,997 (29.9)	248 (0.6)	<0.001
Yes	6,543	490 (7.5)	6,053	46 (0.8)	611 (10.1)	2,892 (47.8)	2,438 (40.3)	66 (1.1)	
Missing	226	41 (18.1)	185	0 (0.0)	23 (12.4)	98 (53.0)	63 (34.1)	1 (0.5)	
**Educational attainment** ^b^									
Junior high school	10,833	3,669 (33.9)	7,164	116 (1.6)	1,091 (15.2)	4,731 (66.0)	1,201 (16.8)	25 (0.3)	<0.001
High school	27,902	3,797 (13.6)	24,105	170 (0.7)	2,612 (10.8)	13,724 (56.9)	7,457 (30.9)	142 (0.6)	
Other	12,923	976 (7.6)	11,947	58 (0.5)	1,100 (9.2)	6,075 (50.8)	4,601 (38.5)	113 (0.9)	
University or more	2,605	89 (3.4)	2,516	13 (0.5)	191 (7.6)	1,168 (46.4)	1,113 (44.2)	31 (1.2)	
Missing	1,040	407 (39.1)	633	8 (1.3)	94 (14.8)	401 (63.3)	126 (19.9)	4 (0.6)	
**Place of residence**									
Yokote (Akita)	14,418	2,784 (19.3)	11,634	75 (0.6)	1,196 (10.3)	6,477 (55.7)	3,791 (32.6)	95 (0.8)	<0.001
Saku (Nagano)	14,813	2,405 (16.2)	12,408	111 (0.9)	1,428 (11.5)	6,893 (55.6)	3,906 (31.5)	70 (0.6)	
Ninohe/Karumai (Iwate)	4,894	952 (19.5)	3,942	28 (0.7)	548 (13.9)	2,232 (56.6)	1,107 (28.1)	27 (0.7)	
Unzen/Minamishimabara (Nagasaki)	5,513	826 (15.0)	4,687	41 (0.9)	410 (8.7)	2,658 (56.7)	1,538 (32.8)	40 (0.9)	
Ozu (Ehime)	3,504	384 (11.0)	3,120	25 (0.8)	363 (11.6)	1,652 (52.9)	1,054 (33.8)	26 (0.8)	
Konan/Aki (Kochi)	3,570	580 (16.3)	2,990	33 (1.1)	323 (10.8)	1,724 (57.7)	891 (29.8)	19 (0.6)	
Chikusei (Ibaraki)	8,591	1,007 (11.7)	7,584	52 (0.7)	820 (10.8)	4,463 (58.8)	2,211 (29.2)	38 (0.5)	

BMI, body mass index; DM, diabetes mellitus; HTN, hypertension.

^a^for smoking status at first pregnancy, “no” combines “never smoke or starting after the 1st pregnancy”;

^b^for educational attainment, “other” means “junior college/specialty/4 year system dropout”;

^c^*P*-value excludes missing data.

The prevalence of PIH and GDM by each category of birth weight, as well as odds ratios for PIH and GDM compared to the reference category (3,000–3,999 g), based on the partially imputed dataset (*n* = 46,365) are described in Table 2. PIH and GDM were observed in 4,048 (8.7%) and 1,867 (4.0%) women, respectively. After adjustment for all possible confounders (model 3b), compared with women born at 3,000–3,999 grams, significantly higher risk of PIH were observed for women born <1,500 grams (adjusted odds ratio [aOR] 1.60; 95% confidence interval [CI], 1.17–2.21), women born 1,500–2,499 grams (aOR 1.16; 95% CI, 1.03–1.30), and women born 2,500–2,999 grams (aOR 1.13; 95% CI, 1.04–1.22). Point estimate of the odds ratio was high for women born over 4,000 grams although we failed to observe a significant effect (aOR 1.41; 95% CI, 0.97–2.04). For GDM, only women born 1,500–2,499 grams had significantly higher risk compared with women born 3,000–3,999 grams, while no significant association was observed for women in other birth weight categories. No substantial differences in estimated effects were observed between the four models including different combinations of possible confounders.

**Table 2.** Odds ratio for PIH and GDM associated with categories of birth weight based on the partially imputed dataset (covariates imputed except for birthweight, *N* = 46,365)

	Birth weight				
	<1,500 g (*n* = 365)	1,500–2,499 g (*n* = 5,088)	2,500–2,999 g (*n* = 26,099)	3,000–3,999 g (*n* = 14,498)	>4,000 g (*n* = 315)
	OR [95% CI]				
**PIH**					
***n* (%)**	46 (12.6)	480 (9.4)	2,346 (9.0)	1,143 (7.9)	33 (10.5)

Model 1^a^	1.60 [1.16;2.19]	1.16 [1.03;1.30]	1.11 [1.03;1.20]	reference	1.41 [0.98;2.04]
Model 2^b^	1.58 [1.15;2.17]	1.13 [1.01;1.27]	1.10 [1.02;1.19]	reference	1.43 [0.99;2.07]
Model 3a^c^	1.57 [1.14;2.15]	1.13 [1.00;1.26]	1.10 [1.02;1.19]	reference	1.43 [0.99;2.07]
Model 3b^d^	1.60 [1.17;2.21]	1.16 [1.03;1.30]	1.13 [1.04;1.22]	reference	1.41 [0.97;2.04]

**GDM**					
***n* (%)**	10 (2.7)	211 (4.1)	945 (3.6)	678 (4.7)	23 (7.3)

Model 1^a^	0.79 [0.42;1.50]	1.18 [1.00;1.38]	1.00 [0.90;1.11]	reference	1.34 [0.87;2.07]
Model 2^b^	0.80 [0.42;1.51]	1.18 [1.00;1.39]	1.01 [0.91;1.13]	reference	1.28 [0.83;1.98]
Model 3a^c^	0.79 [0.41;1.49]	1.18 [1.00;1.39]	1.03 [0.92;1.14]	reference	1.26 [0.81;1.95]
Model 3b^d^	0.80 [0.42;1.52]	1.20 [1.02;1.42]	1.04 [0.94;1.16]	reference	1.24 [0.80;1.93]

CI, confidence interval; GDM, gestational diabetes mellitus; OR, odds ratio; PIH, pregnancy-induced hypertension.

^a^model 1: adjusted for birth year.

^b^model 2: model 1 + (education) + (family history) + (passive smoking at 10 years old) + (height) + (older sibling).

^c^model 3a: model 2 + (age at first pregnancy) + (age at first pregnancy) × (birth year) + (smoking status).

^d^model 3b: model 3a + (BMI at 20 years old).

When we further conducted the same analysis including complete data only, estimated effect of birth weight on risk of pregnancy outcomes were similar to the result of main analyses (eTable 2). When we conducted the same analysis on the fully imputed dataset, we observed attenuation of the association to some degree compared to the main analysis, although the associations remained significant (eTable 3).

## DISCUSSION

In the current study, we observed that women born with lower birth weight had significantly increased risk of PIH during their own pregnancy. Although the risk of GDM was significantly high in women born to 1,500–2,499 grams than those born to 3,000–3,999 grams, we failed to observe significant effects among other categories.

In line with previous studies,^5^^–^^9^ we observed a significant association between birth weight and their risk for PIH. The phenomenon is understandable in general as women born low birth weight have higher risk for chronic hypertension in later life.^1^^,^^3^^,^^4^ The exact mechanism as to why lower birth weight may increase the risk of PIH has not yet been identified, however, several possible pathways have been proposed. One possible mechanism is through impaired endothelial function resulting in elevated von Willebrand factor,^24^ leading to elevation of blood pressure. Another mechanism proposed from previous studies is through elevated plasma insulin level and reduced glucose tolerance.^25^^,^^26^ A third mechanism is through low nephron formation^27^ among those born with low birth weight leading to reduced renal function.

The association between macrosomia and future hypertension requires future research. A previous meta-analysis found women born macrosomia are at higher risk of hypertension at adolescence but at reduced risk for hypertension as an adult.^28^ A previous study on pregnant women found that women born macrosomia were at elevated risk of PIH.^5^ In our study although we observed elevated risk, the results were not statistically significant. As our study was limited with small sample of women born over 4,000 g (only 348 [0.6%]), further larger studies regarding the effect between higher birthweight and PIH risk are anticipated.

While previous studies in Europe and United States showed increased risk for GDM among women with lower birth weight,^9^^,^^10^^,^^12^ we failed to detect a significant increase in risk for women born at lower birth weight (<1,500 g) compared to women born at 3,000–3,999 g, although we detected a significantly higher risk for GDM among women born 1,500–2,499 g. One plausible reason could be the small number of women in this category (only 10 had GDM among 365 women born under 1,500 grams) and lack of power to detect a true association. Further research on larger samples is anticipated.

Our study utilized a large population cohort, allowing us to undertake a rigorous analysis that considered a broad range of possible confounders while maintaining adequate statistical power. To our knowledge, this is the largest study examining the association between birth weight and subsequent risk of pregnancy complications among Asian women. Nonetheless, this study had limitations. First, information on women’s own gestational age at birth was not available. Low birth weight can be due to shorter gestation and/or smaller fetal growth, and recent studies suggest their influences on future health may differ.^8^^,^^10^^,^^12^ Future studies investigating the individual contributions of shorter gestation and smaller fetal growth on risk of pregnancy complications are warranted. Second, birth weight was collected in a retrospective manner and was self-reported. While a small study in Japan suggested self-reported birth weight categories may correlate with actual birth weight,^29^ larger studies in other countries have shown that self-reported birth weight tends to be inaccurate and thus could cause recall bias, so self-reported birth weight should be used with caution.^30^^,^^31^ Furthermore, we retrieved pregnancy characteristics, including PIH and GDM, in a retrospective manner as well. Although some studies have showed the validity of self-reported GDM and PIH,^32^^,^^33^ this could have led to misclassification. These misclassification issues of both the exposure and outcomes are likely to be non-differential in nature and may have resulted in an underestimate of the association. Additionally, clinically standardized definitions of GDM and PIH have undergone minor changes. As for PIH, from 1986 to 2005, PIH was defined as women with one or more of the following symptoms between 20 weeks of gestation and 6 weeks postpartum; hypertension (≥140 mm Hg), proteinuria, and edema. In 2005, a modified definition was implemented as follows; women who developed hypertension (≥140 mm Hg) or those with chronic hypertension who developed proteinuria, between 20 weeks of gestation and 6 weeks postpartum. As for GDM, no national guideline to screen or diagnose GDM existed until 1984, when the Japan Society of Obstetrics and Gynecology recommended the use of a 75 g oral glucose tolerance test to diagnose GDM according to one or more of the following criteria; fasting blood sugar >100 mg/dL, 1-hour 75 g oral glucose tolerance test (OGTT) >180 mg/dL, and 2-hour 75 g OGTT >150 mg/dL. Studies that utilize birth weights and pregnancy complications obtained by potentially more accurate methods, such as official records by healthcare provider, may help to add clarity to this area of investigation.

In conclusion, among a large population of Japanese women, we observed that those born at lower birth weights had a subsequent increased risk of PIH during their own pregnancy.

## APPENDIX A. SUPPLEMENTARY DATA

eTable 1. Distribution of characteristics based on the dataset which exclude women with any missing data (complete data; *n* = 41,285) and the dataset which impute all variables (fully imputed; *n* = 55,303)

eTable 2. Odds ratio for each outcome based on complete cases dataset (*N* = 41,285)

eTable 3. Odds ratio for each outcome based on the dataset which impute all variables (*N* = 55,303)
