# Supplementary material for: Association Between Birth Weight and Risk of Pregnancy-Induced Hypertension and Gestational Diabetes in Japanese Women: JPHC-NEXT Study
Source: J Epidemiol. 2022 Apr 5;32(4):168–73. doi: 10.2188/jea.JE20200302 (PMC8918619; doi:10.2188/jea.JE20200302)
Supplement: Supplementary file 1 [file je-32-168-s001.pdf]

eTable 1. Distribution of characteristics based on the dataset which exclude women with any missing data (complete data; n=41,285) and the dataset which impute all variables (fully imputed; n=55,303)

	Birth weight									
	<1,500 g		1,500–2,499 g		2,500–2,999 g		3,000–3,999 g		>4,000 g	
**n (%)**	Complete data[a]	Fully imputed[b]	Complete data[a]	Fully imputed[b]	Complete data[a]	Fully imputed[b]	Complete data[a]	Fully imputed[b]	Complete data[a]	Fully imputed[b]
**Age at first pregnancy**										
15–24	121 (40.2)	213 (45.0)	1,763 (39.9)	2,878 (43.7)	9,206 (39.8)	13,388 (42.5)	4,314 (32.8)	5,781 (35.4)	76 (27.0)	103 (29.2)
25–29	138 (45.8)	212 (44.8)	2,051 (46.4)	2,927 (44.4)	10,909 (47.2)	14,418 (45.7)	6,309 (47.9)	7,647 (46.8)	127 (45.0)	160 (45.1)
30–34	32 (10.6)	37 (7.9)	466 (10.5)	604 (9.2)	2,321 (10.0)	2,887 (9.2)	1,857 (14.1)	2,147 (13.1)	57 (20.2)	67 (18.9)
>34	10 (3.3)	11 (2.3)	138 (3.1)	181 (2.7)	677 (2.9)	840 (2.7)	691 (5.2)	777 (4.8)	22 (7.8)	24 (6.8)
**BMI at 20 years**										
<18.5	58 (19.3)	75 (15.8)	747 (16.9)	1,011 (15.3)	2,965 (12.8)	3,827 (12.1)	1,623 (12.3)	1,902 (11.6)	32 (11.3)	37 (10.5)
18.5–24.9	224 (74.4)	360 (76.0)	3,422 (77.5)	5,156 (78.2)	18,992 (82.2)	25,874 (82.1)	10,764 (81.7)	13,389 (81.9)	231 (81.9)	289 (81.7)
≥25.0	19 (6.3)	39 (8.2)	249 (5.6)	423 (6.4)	1156 (5.0)	1,831 (5.8)	784 (6.0)	1,062 (6.5)	19 (6.7)	28 (7.8)
**Current height**										
< 151	123 (40.9)	228 (48.1)	1,610 (36.4)	2,762 (41.9)	5,821 (25.2)	9,053 (28.7)	1,416 (10.8)	2,114 (12.9)	14 (5.0)	26 (7.2)
151–155	103 (34.2)	147 (31.1)	1,398 (31.6)	2,028 (30.8)	7,566 (32.7)	10,326 (32.7)	3,325 (25.2)	4,284 (26.2)	42 (14.9)	55 (15.6)
156–159	47 (15.6)	62 (13.2)	856 (19.4)	1,125 (17.1)	5,604 (24.2)	7,153 (22.7)	3,773 (28.6)	4,575 (28.0)	84 (29.8)	101 (28.7)
≥160	28 (9.3)	36 (7.6)	554 (12.5)	675 (10.2)	4,122 (17.8)	5,000 (15.9)	4,657 (35.4)	5,380 (32.9)	142 (50.4)	171 (48.4)
**passive smoking at 10 years**										
Almost none	150 (49.8)	248 (52.5)	2,230 (50.5)	3,479 (52.8)	11,929 (51.6)	16,696 (52.9)	6,265 (47.6)	7,964 (48.7)	126 (44.7)	161 (45.5)
1–3 times a month	8 (2.7)	13 (2.7)	186 (4.2)	249 (3.8)	997 (4.3)	1,268 (4.0)	607 (4.6)	709 (4.3)	19 (6.7)	21 (5.8)
1–4 times a week	27 (9.0)	37 (7.7)	325 (7.4)	452 (6.9)	2014 (8.7)	2,558 (8.1)	1,215 (9.2)	1,430 (8.7)	24 (8.5)	29 (8.2)
Almost every day	116 (38.5)	175 (37.1)	1,677 (38.0)	2,409 (36.6)	8,173 (35.4)	11,011 (34.9)	5,084 (38.6)	6,250 (38.2)	113 (40.1)	143 (40.5)
**Smoking status at first pregnancy[c]**										

No	258 (85.7)	424 (89.6)	3,855 (87.3)	5,902 (89.6)	20,433 (88.4)	28,264 (89.6)	10,875 (82.6)	13,724 (83.9)	225 (79.8)	283 (79.9)
Yes	43 (14.3)	49 (10.4)	563 (12.7)	688 (10.4)	2,680 (11.6)	3,269 (10.4)	2,296 (17.4)	2,629 (16.1)	57 (20.2)	71 (20.1)
**Educational attainment[d]**										
Junior high school	87 (28.9)	182 (38.5)	866 (19.6)	1,911 (29.0)	3,864 (16.7)	7,182 (22.8)	962 (7.3)	1,858 (11.4)	21 (7.4)	36 (10.3)
High school	145 (48.2)	211 (44.5)	2,340 (53.0)	3,241 (49.2)	12,493 (54.1)	16,343 (51.8)	6,846 (52.0)	8,443 (51.6)	127 (45.0)	164 (46.2)
Other	56 (18.6)	67 (14.2)	1,026 (23.2)	1,235 (18.7)	5,638 (24.4)	6,768 (21.5)	4,291 (32.6)	4,905 (30.0)	103 (36.5)	121 (34.3)
University or more	13 (4.3)	13 (2.8)	186 (4.2)	204 (3.1)	1,118 (4.8)	1,240 (3.9)	1,072 (8.1)	1,147 (7.0)	31 (11.0)	33 (9.2)

[a] "Fully imputed": based on the dataset of all women who gave birth with all missing variables imputed;

[b] "Partially imputed": based on the dataset of all women who gave birth and had self-reported birthweight, and with all missing variables except for birthweight imputed;

[c] Smoking status at first pregnancy, "No" combines "Never smoke or starting after the 1st pregnancy";

[d] Educational attainment, "Other" means "Junior college / specialty / 4 year system dropout"

eTable 2. Odds ratio for each outcome based on complete cases dataset (N=41,285)

	**Birth weight**				
	<1,500 g (n=301)	1,500–2,499 g (n=4,418)	2,500–2,999 g (n=23,113)	3,000–3,999 g (n=13,171)	>4,000 g (n=282)
**Pregnancy induced hypertension**					
n(%)	42 (14.0)	411 (9.3)	2,025 (8.8)	1,018 (7.7)	27 (9.6)
Odds ratio [95% confidence interval][a]					
Model 1	1.82 [1.30;2.54]	1.16 [1.03;1.31]	1.10 [1.02;1.20]	reference	1.32 [0.88;1.97]
Model 2	1.81 [1.30;2.54]	1.14 [1.01;1.29]	1.09 [1.01;1.19]	reference	1.34 [0.89;2.01]
Model 3a	1.79 [1.28;2.51]	1.14 [1.00;1.29]	1.09 [1.01;1.19]	reference	1.34 [0.90;2.01]
Model 3b	1.85 [1.32;2.59]	1.17 [1.03;1.32]	1.11 [1.03;1.21]	reference	1.33 [0.88;1.99]
**Gestational diabetes mellitus**					
n(%)	8 (2.7)	189 (4.3)	830 (3.6)	617 (4.7)	19 (6.7)
Odds ratio [95% confidence interval][a]					
Model 1	0.75 [0.37;1.52]	1.20 [1.01;1.42]	0.98 [0.88;1.10]	reference	1.22 [0.75;1.96]
Model 2	0.75 [0.37;1.52]	1.20 [1.01;1.43]	1.00 [0.89;1.12]	reference	1.16 [0.72;1.87]
Model 3a	0.73 [0.36;1.49]	1.20 [1.01;1.43]	1.01 [0.90;1.13]	reference	1.15 [0.71;1.85]
Model 3b	0.75 [0.37;1.53]	1.22 [1.03;1.46]	1.03 [0.92;1.15]	reference	1.14 [0.70;1.84]

[a] Multilevel logistic regression model with place of residence as random effect
Model 1: birth year
Model 2: + (education) + (family history) + (passive smoking at 10 years old) + (height) + (older sibling)
Model 3a: + (age at first pregnancy) + (age at first pregnancy) x (birth year) + (smoking status)
Model 3b: + (BMI at 20 years old)

eTable 3. Odds ratio for each outcome based on the dataset which impute all variables (N=55,303)

	Birth weight				
	<1,500g (n=491)	1,500–2,499 g (n=6,613)	2,500–2,999 g (n=31,462)	3,000–3,999 g (n=16,389)	>4,000 g (n=348)
**Pregnancy induced hypertension**					
n (%)	58 (11.9)	627 (9.5)	2841 (9.0)	1305 (8.0)	36 (10.2)
Odds ratio [95% confidence interval][a]					
Model 1	1.45 [1.07;1.98]	1.15 [1.03;1.29]	1.11 [1.02;1.20]	reference	1.36 [0.95;1.94]
Model 2	1.45 [1.06;1.97]	1.14 [1.01;1.27]	1.09 [1.01;1.19]	reference	1.36 [0.95;1.96]
Model 3a	1.44 [1.05;1.97]	1.13 [1.01;1.27]	1.10 [1.01;1.19]	reference	1.37 [0.96;1.96]
Model 3b	1.56 [1.14;2.14]	1.16 [1.03;1.30]	1.12 [1.03;1.22]	reference	1.37 [0.94;1.99]
**Gestational diabetes mellitus**					
n(%)	13 (2.8)	246 (3.7)	1068 (3.4)	723 (4.4)	24 (6.8)
Odds ratio [95% confidence interval][a]					
Model 1	0.84 [0.46;1.57]	1.15 [0.98;1.36]	1.00 [0.90;1.11]	reference	1.34 [0.87;2.05]
Model 2	0.85 [0.46;1.59]	1.15 [0.97;1.37]	1.01 [0.91;1.13]	reference	1.29 [0.84;1.98]
Model 3a	0.85 [0.46;1.58]	1.15 [0.98;1.36]	1.03 [0.92;1.14]	reference	1.27 [0.82;1.96]
Model 3b	0.87 [0.47;1.61]	1.19 [1.01;1.41]	1.04 [0.93;1.16]	reference	1.22 [0.78;1.93]

[a] Multilevel logistic regression model with place of residence as random effect

Model 1: birth year

Model 2: + (education) + (family history) + (passive smoking at 10 years old) + (height) + (older sibling)

Model 3a: + (age at first pregnancy) + (age at first pregnancy) x (birth year) + (smoking status)

Model 3b: + (BMI at 20 years old)
